# Associations between Oral Human Herpesvirus-6 and -7 and Periodontal Conditions in Older Adults

**DOI:** 10.3390/life13020324

**Published:** 2023-01-23

**Authors:** Natsuki Hamada, Hideo Shigeishi, Iori Oka, Mio Sasaki, Honami Kitasaki, Mariko Nakamura, Kanako Yano, Chia-Hsin Wu, Yoshino Kaneyasu, Tomoko Maehara, Masaru Sugiyama, Kouji Ohta

**Affiliations:** Department of Public Oral Health, Program of Oral Health Sciences, Graduate School of Biomedical and Health Sciences, Hiroshima University, Hiroshima 734-8553, Japan

**Keywords:** human herpesvirus-7, real-time polymerase chain reaction, periodontitis, older adults

## Abstract

**Simple Summary:**

The objective of this study was to uncover the association between oral human herpesvirus-6 (HHV-6) and HHV-7 infection and periodontitis. Oral HHV-6 and HHV-7 DNA were analyzed by a real-time polymerase chain reaction using tongue swab samples from 74 older adults. Of the 74 participants, one participant (1.4%) was HHV-6 DNA-positive and 36 participants (48.6%) were HHV-7 DNA-positive. HHV-7 DNA-positive participants had a higher prevalence of diabetes (16.7%) than HHV-7 DNA-negative participants (7.9%). However, there was no significant association between HHV-7 DNA positivity and diabetes. Oral HHV-7 infection was significantly associated with a deep periodontal pocket (*p* = 0.04). HHV-7 DNA-positive participants had a higher positive rate of a periodontal pocket with BOP (25.0%) than the HHV-7 DNA-negative participants (7.9%). Additionally, the HHV-7 DNA-positive participants had higher PISA values (52.7 mm^2^) than the HHV-7 DNA-negative participants (47.2 mm^2^). These results indicate that oral HHV-7 infection is associated with active periodontitis. However, there was no statistically significant association between HHV-7 and a ≥6-mm periodontal pocket with BOP positivity or the PISA value. Oral herpesviruses other than HHV-7 may be more closely associated with periodontitis. It is necessary to clarify the association between HHV-7 and periodontitis with the detection of HHV-7 in the periodontal pocket.

**Abstract:**

Background: The associations between oral human herpesvirus-6 (HHV-6) and HHV-7, periodontal conditions, and lifestyle-related diseases, such as hypertension, diabetes, and dyslipidemia, have not been fully investigated in older adults. Methods: Seventy-four older patients who visited Hiroshima University Hospital were enrolled. Tongue swab samples were employed, and a real-time polymerase chain reaction was performed to detect HHV-6 and HHV-7 DNA. Dental plaque accumulation, probing pocket depth, and bleeding on probing (BOP) (i.e., a sign of periodontal inflammation) were examined. The periodontal inflamed surface area (PISA) value (i.e., an indicator of the severity of periodontitis) was also examined. Results: Of the 74 participants, one participant (1.4%) was HHV-6 DNA-positive and 36 participants (48.6%) were HHV-7 DNA-positive. A significant association between HHV-7 DNA and probing depth was found (*p* = 0.04). The HHV-7 DNA-positive participants had a higher positive rate of a ≥6-mm periodontal pocket with BOP (25.0%) than the HHV-7 DNA-negative participants (7.9%). Additionally, the HHV-7 DNA-positive participants had a higher PISA value than the HHV-7 DNA-negative participants. However, there was no significant association between HHV-7 and the PISA value (*p* = 0.82). No significant association was found between HHV-7 and lifestyle-related diseases (*p* > 0.05). Conclusions: Oral HHV-7 infection is associated with a deep periodontal pocket.

## 1. Introduction

The oral cavity is a common latent infection site for human herpesviruses. Oral herpesviruses and periodontopathic bacteria are highly involved in periodontal inflammation [1,2]. Double infection of oral human cytomegalovirus (HCMV) and *Porphyromonas gingivalis* was associated with the presence of deep periodontal pockets with bleeding in Japanese adults, suggesting that HCMV is involved in active periodontitis with periodontopathic bacteria [3]. Additionally, oral Epstein–Barr virus (EBV) infection was associated with oral hygiene and the severity of periodontal inflammation [4]. Increased dental plaque accumulation was found in oral EBV-positive participants [4]. Furthermore, a significant relationship has been found between double infection of oral EBV and *Porphyromonas gingivalis* and diabetes [4]. Attenuated immune responses in diabetic patients may be related to the activation of herpesviruses.

Human herpesvirus (HHV)-6A, HHV-6B, and HHV-7 are members of the Roseolovirus genus of the β-herpesviruses subfamily [5]. HHV-6, which was first isolated from HIV-infected patients [6], is closely associated with multiple sclerosis, encephalitis, and cancer [7,8]. HHV-7 was first isolated from CD4+ T cells in the peripheral blood of healthy individuals [9]. Primary infection of HHV-7 causes exanthem subitem (i.e., common febrile exanthematous illness) in infants and young children [10]. HHV-7 latently infects CD4+ T cells and epithelial cells in the salivary glands [11,12]. HHV can reactivate in response to host immunosuppression and an inflammatory stimulus [9]. Therefore, it has been hypothesized that reactivation of HHV may be associated with oral chronic inflammatory diseases such as periodontitis. However, an association between oral HHV-6 and HHV-7 and periodontal inflammation has not been elucidated in older adults. Additionally, a relationship between oral HHV-6 and HHV-7 and lifestyle-related diseases, such as hypertension, diabetes, and dyslipidemia remains unknown. The objective of this study was to uncover the associations between oral HHV-6 and -7 infection and periodontitis or lifestyle-related diseases in Japanese older adults.

## 2. Materials and Methods

### 2.1. Participants

Seventy-four participants who visited Hiroshima University Hospital from April 2021 to September 2021, ranging in age from 62 to 86 with a median age of 73, were enrolled in this study. The Ethical Committee of Hiroshima University approved the study protocol (approval no. E2017-1115) and each participant agreed to participate in this study and signed an informed consent form. Regarding participants’ clinical characters, denture use and the number of remaining teeth were recorded. In addition, lifestyle-related diseases such as hypertension, diabetes, and dyslipidemia were recorded. Hypertension was defined as having high blood pressure (≥140/90 mm Hg) or taking anti-hypertensive medications [4]. Diabetes was defined as having a high hemoglobin A1c level (≥6.5%) or taking anti-diabetic medications [4]. Dyslipidemia was defined as taking anti-hyperlipidemic medications or having high levels of triglyceride (≥150 mg/dL), high levels of low-density lipoprotein cholesterol (≥140 mg/dL), or low levels of high-density lipoprotein cholesterol (<40 mg/dL) [4].

### 2.2. Collection of Oral Samples and DNA Extraction

Specimens were collected by swabbing the tongue’s surface with a sterile disposable brush (Orcellex^®^ Brush; Rovers Medical Devices, Oss, The Netherlands). The tongue’s surface was non-invasively swabbed approximately 10 times using an Orcellex^®^ Brush. The brush was soaked and washed in 800 μL cell lysis buffer (Invitrogen; Thermo Fisher Scientific Inc., Waltham, MA, USA) in a 15-mL plastic tube immediately after swabbing the tongue’s surface. Collected samples dissolved in lysis buffer in plastic tubes were stored at −80 °C until further processing. DNA extraction was performed using a PureLink™ Microbiome DNA Purification Kit (Invitrogen) in accordance with manufacturer’s protocol.

### 2.3. Assessment of Periodontal Conditions and Dental Plaque Accumulation

The probing pocket depth and bleeding on probing (BOP) were examined at all remaining teeth in each participant to evaluate periodontal health. Probing depth was investigated at six sites (buccal, lingual, mesiobuccal, mesiolingual, distobuccal, and distolingual). The PISA value (i.e., the surface area of the periodontal pocket with bleeding) and periodontal epithelial surface area (PESA) value (i.e., total surface area of the periodontal pocket) were obtained in accordance with a previous study [13]. The PISA value is employed as an indicator of the severity of periodontitis. To evaluate oral hygiene conditions, the degree of dental plaque accumulation was examined using plaque control record scores in accordance with a previous study [4].

### 2.4. Real-Time PCR Analysis

Real-time PCR was conducted using a Thermal Cycler Dice^®^ Real Time System III (Takara, Osaka, Japan) to detect HHV-7 DNA. The reaction mixture included FastGene™ QPCR Probe Mastermix w/ROX (10 µL, Nippon Genetics Co., Ltd., Tokyo, Japan), PCR primers (250 nM, Hokkaido System Science Co., Ltd., Sapporo, Japan) and PCR probe (500 nM, Hokkaido System Science Co., Ltd.). The real-time PCR amplification protocol was as follows: 95 °C for 10 min and then 50 cycles of 95 °C for 15 s, 60 °C for 60 s, and 40 °C for 30 s. The primer sequences for HHV-6 were 5′-TTTGCAGTCATCACGATCGG-3′ (forward) [14] and 5′-AGAGCGACAAATTGGAGGTTTC-3′ (reverse) [14]. The probe sequence for HHV-6 was 5′-FAM-AGCCACAGCAGCCATCTACATCTGTCAA-TAMRA-3′ [14]. Serial 10-fold dilutions of an HHV-6 positive DNA sample (580–5.8 ng/μL) were employed to construct a standard curve (Supplementary Figure S1). The primer sequences for HHV-7 were 5′-TCCAGAAATGATAGACAGATGTTGGT-3′ (forward) [15] and 5′-CATGGGCACATTTGTACTTCAAAG-3′ (reverse) [15]. The probe sequence for HHV-7 was 5′-FAM-AGCTATCCTAATGAAGGCT-TAMRA-3′ [15]. Serial 10-fold dilutions of an HHV-7 positive DNA sample (25.0–0.025 ng/μL) were employed to construct a standard curve (Supplementary Figure S2).

### 2.5. Statistical Analysis

Fisher’s exact probability test or the χ^2^ test was conducted to assess significant associations between nominal variables and HHV-6 or HHV-7. The Mann–Whitney U-test was conducted to evaluate significant differences in continuous variables between two independent groups. Logistic regression analysis with forced entry was conducted to examine the relationship between HHV-7 as a dependent variable and clinical variables with a *p*-value of < 0.2 through univariate analysis as an independent variable. *p* < 0.05 was considered statistically significant.

## 3. Results

### 3.1. Relationship between Oral HHV-6 and HHV-7 and Clinical Parameters

HHV-6 DNA was positive in one of 74 participants (1.4%). A summary of the association between HHV-6 DNA and clinical factors is shown in Table 1. The association between HHV-6 DNA and clinical factors remains unclear due to the low prevalence of oral HHV-6. HHV-7 DNA was positive in 36 of 74 participants (48.6%). A summary of the association between HHV-7 DNA and clinical factors is shown in Table 2. Participants in their 70s had a higher HHV-7 DNA-positive rate (56.3%) than participants in their 60s (40%) and 80s (47.1%). Male participants had a higher HHV-7 DNA-positive rate (66.7%) than female participants (41.5%), but a significant difference was not found (*p* = 0.71). HHV-7 DNA-positive participants had a lower median number of remaining teeth (22 teeth) and a higher percentage of denture use (44.4%) than HHV-7 DNA-negative participants (25 teeth and 34.2%). HHV-7 DNA-positive participants had a higher prevalence of diabetes (16.7%) and dyslipidemia (25.0%) than HHV-7 DNA-negative participants (7.9% and 13.2%). However, there was no significant association between HHV-7 DNA positivity and diabetes or dyslipidemia (*p* = 0.30, *p* = 0.24).

### 3.2. Relationship between Oral HHV-6 and HHV-7 and Periodontal Conditions

A summary of the association between HHV-6 DNA and periodontal conditions is shown in Table 3. The HHV-6 DNA-positive participant had a ≥ 6-mm periodontal pocket without BOP. Associations between oral HHV-7 and dental plaque accumulation and periodontal conditions are summarized in Table 4. A significant association between HHV-7 DNA and plaque control record scores was not found (*p* = 0.27). Conversely, a significant association between HHV-7 DNA and the probing depth was found (*p* = 0.04). HHV-7 DNA-positive participants had a higher positive rate of a ≥ 6-mm periodontal pocket with BOP (25.0%) than HHV-7 DNA-negative participants (7.9%). Additionally, HHV-7 DNA-positive participants had a higher median PISA value than HHV-7 DNA-negative participants. However, there was no significant difference between HHV-7 DNA and a ≥ 6-mm periodontal pocket with BOP positivity or the PISA value (*p* = 0.06, *p* = 0.82).

### 3.3. Logistic Regression Analysis with HHV-7 as a Dependent Variable

Next, the association between HHV-7 and clinical parameters was examined by logistic regression analysis. Logistic regression analysis with HHV-7 as a dependent variable and variables with a *p*-value of < 0.2 in univariate analysis (i.e., the number of remaining teeth, the probing depth, and a ≥6-mm periodontal pocket with BOP) as independent variables was performed. The results of this logistic regression analysis are summarized in Table 5. There was no significant association between HHV-7 and the number of remaining teeth, the probing depth, or a ≥6-mm periodontal pocket with BOP.

## 4. Discussion

HHV-7 is primarily transmitted through saliva and breast milk [16]. The common clinical features of HHV-7 primary infection in infants and children include exanthem subitem, high fever, and central nervous disorder [10,17]. Exanthem subitem is marked by a sudden and high fever for 3–5 days, followed by a skin rash [10,17]. HHV-7 is associated with hematopoietic stem cell transplantation-related oral mucositis and salivary gland malignant tumors [18,19], indicating that some diseases are associated with reactivation of HHV-7. The oral HHV-7-positive rate increased after renal transplantation compared with the pretransplant period in renal transplant recipients, suggesting that latent HHV-7 is reactivated under immunosuppressive conditions [20].

In terms of oral herpesvirus infection, the prevalence of oral HHV-7 (61.5%) is likely to be higher than that of oral EBV (7.7%) and oral HCMV (7.7%) in subjects without periodontitis [21]. Cassai et al., reported that HHV-7 DNA was detected in gingival biopsy specimens from 10 of 13 patients with chronic periodontitis (77%) and 7 of 10 healthy subjects (70%) [22]. Additionally, HHV-7 DNA was detected in both affected and non-affected sites in periodontitis patients [22]. However, the prevalence of oral HHV-6 was low in both chronic periodontitis patients and healthy subjects [22]. These results suggest that HHV-7 is commonly found in periodontal tissue and HHV-6 is rarely found in the oral cavity. However, HHV-7 was more frequently detected in participants with chronic or aggressive periodontitis (92%) compared with participants without chronic and aggressive periodontitis (62%), suggesting an association between HHV-7 and periodontal inflammation [21]. In this study, oral HHV-7-positive participants had a higher PISA value (i.e., severity of the periodontitis) than HHV-7-negative participants, suggesting that oral HHV-7 can be reactivated by periodontal inflammation. However, a significant association between HHV-7 and a deep periodontal pocket with bleeding (i.e., active periodontitis) and the PISA value was not found in this study. Thus, it is controversial whether HHV-7 is closely associated with periodontitis. We had previously found that EBV and CMV were significantly associated with the presence of a deep periodontal pocket with bleeding [3,23]. Therefore, oral herpesviruses such as EBV and HCMV, rather than HHV-7, may be more closely associated with active periodontitis.

Coinfection of EBV and *Porphyromonas gingivalis* was associated with a high PISA value in patients with periodontitis [4], indicating that oral herpesviruses and periodontopathic bacteria may be cooperatively involved in periodontitis. *P. gingivalis*-produced butyric acid contributes to the reactivation of EBV by enhancing transcriptional activity of EBV BZLF1 [24]. Therefore, *P. gingivalis* has been implicated in EBV reactivation. Reactivated EBV enhances the release of interleukins from gingival fibroblasts and inhibits macrophage phagocytosis [25,26]. Therefore, *P. gingivalis*-activated EBV might play a critical role in periodontitis pathology. It has been speculated that both oral herpesviruses and periodontopathic bacteria are involved in periodontitis progression by inhibiting the host immune response and enhancing periodontal inflammation. However, a relationship between coinfection of HHV-7 and periodontopathic bacteria and periodontitis remains unknown.

As for the association between herpesvirus infection and lifestyle-related diseases, diabetic patients had an increased risk of EBV infection [27]. No significant relationship between oral HHV-6 and -7 infection and lifestyle-related diseases was found in this study. However, HHV-7-positive participants had a higher prevalence of diabetes compared with HHV-7-negative participants. Attenuated immune responses in diabetic patients may be related to HHV-7 infection in the oral cavity. Further additional study will be required to clarify the relationship between oral HHV-7 infection and diabetes.

It is thought that most people with HHV-6 were infected in early childhood [28]. HHV-6 establishes latent infection in lymphocytes and can be reactivated under immunosuppressive conditions [29]. Additionally, HHV-6 may be involved in cancer development [7]. In this study, oral HHV-6 positivity was rare in older adults, suggesting that its prevalence may be low in older adults. However, the prevalence of oral HHV-6 in young and middle-aged people remains unknown.

Swab samples of the tongue’s dorsum were collected using a soft brush in this study. Tongue swab samples contain oral microorganisms, tongue epithelial cells, and saliva. It was believed that salivary HHV-7 was detected using tongue swab samples in this study. The inflammatory periodontal pocket is a reservoir of herpesviruses in the oral cavity [1]. However, it remains unclear whether HHV-7 had infected periodontal tissues in this study. Further investigation of HHV-7 in biopsy samples from the periodontal pocket, subgingival dental plaque, and gingival crevicular fluid is required to clarify the presence of HHV-7 in the periodontal pocket.

This study had some limitations. First, because this study enrolled older participants only, oral HHV-6 and HHV-7 prevalence in young and middle-aged adults remains unknown. Second, the matching of confounding factors (i.e., age, sex, general health condition) was not performed in the HHV-7-positive and -negative cases because of the small number of participants. Finally, the presence of HHV-7 in periodontal tissues remains unclear.

## 5. Conclusions

The prevalence of oral HHV-6 infection was low in older adults. HHV-7 was found in the oral cavity in approximately 50% of older adults. Periodontal pockets may be an important reservoir of HHV-7 in the oral cavity of older adults. However, it remains unclear whether HHV-7 was closely associated with periodontitis in this study. Oral herpesviruses other than HHV-7 may be predominantly involved in active periodontitis. Further study is required to clarify the relationship between HHV-7 and periodontitis by detection of HHV-7 in the inflammatory periodontal pocket.

## Figures and Tables

**Table 1:** Association between oral HHV-6 and clinical parameters.

Variables	HHV-6		*p*-Value
	Negative (*n* = 73)	Positive (*n* = 1)	
Age, median (IQR)	73 (11)	82	0.32 ^a^
Age group, *n* (%)			
60–69	25 (34.2%)	0 (0%)	0.18 ^b^
70–79	32 (43.8%)	0 (0%)	
80–89	16 (21.9%)	1 (100%)	
Gender, *n* (%)			
Male	20 (27.4%)	1 (100%)	0.18 ^b^
Female	53 (72.6%)	0 (0%)	
Hypertension, *n* (%)	17 (23.3%)	0 (0%)	>0.99 ^c^
Diabetes, *n* (%)	8 (11%)	1 (100%)	0.12 ^c^
Dyslipidemia, *n* (%)	13 (17.8%)	1 (100%)	0.19 ^c^
The number of remaining teeth, median (IQR)	24 (7)	14	0.22 ^a^
Denture user, *n* (%)	28 (38.4%)	1 (100%)	0.39 ^c^

IQR: Interquartile range. ^a^ Mann–Whitney U test. ^b^ χ^2^ test. ^c^ Fisher’s exact test.

**Table 2:** Association between oral HHV-7 and clinical parameters.

Variables	HHV-7		*p*-Value
	Negative (*n* = 38)	Positive (*n* = 36)	
Age, median (IQR)	72 (12.3)	74 (10.8)	0.21 ^a^
Age group, *n* (%)			
60–69	15 (39.5%)	10 (27.8%)	0.48 ^b^
70–79	14 (36.8%)	18 (50.0%)	
80–89	9 (23.7%)	8 (22.2%)	
Gender, *n* (%)			
Male	7 (18.4%)	14 (38.9%)	0.71 ^c^
Female	31 (81.6%)	22 (61.1%)	
Hypertension, *n* (%)	10 (26.3%)	7 (19.4%)	0.58 ^c^
Diabetes, *n* (%)	3 (7.9%)	6 (16.7%)	0.30 ^c^
Dyslipidemia, *n* (%)	5 (13.2%)	9 (25.0%)	0.24 ^c^
The number of remaining teeth, median (IQR)	25 (4.3)	22 (7.5)	0.12 ^a^
Denture user, *n* (%)	13 (34.2%)	16 (44.4%)	0.48 ^c^

IQR: Interquartile range. ^a^ Mann–Whitney U test. ^b^ χ^2^ test. ^c^ Fisher’s exact test.

**Table 3:** Association between oral HHV-6 and periodontal conditions.

Variables	HHV-6		*p*-Value
	Negative (*n* = 73)	Positive (*n* = 1)	
Plaque control record scores (%), median (IQR)	14 (14)	33	0.30 ^a^
Probing depth, *n* (%)			
<4 mm	15 (20.5%)	0 (0%)	0.33 ^b^
≥4 mm and <6 mm	36 (49.3%)	0 (0%)	
≥6 mm	22 (30.1%)	1 (100%)	
≥4 mm periodontal pocket with BOP, *n* (%)	34 (46.6%)	0 (0%)	>0.99 ^c^
≥6 mm periodontal pocket with BOP, *n* (%)	12 (16.4%)	0 (0%)	>0.99 ^c^
PISA (mm^2^), median (IQR)	50.9 (102.4)	25.5	0.70 ^a^
PESA (mm^2^), median (IQR)	996.2 (417.3)	575.2	0.30 ^a^

IQR: Interquartile range. PISA: periodontal inflamed surface area. PESA: periodontal epithelial surface area. ^a^ Mann–Whitney U test. ^b^ χ^2^ test. ^c^ Fisher’s exact test.

**Table 4:** Association between oral HHV-7 and periodontal conditions.

Variables	HHV-7		*p*-Value
	Negative (*n* = 38)	Positive (*n* = 36)	
Plaque control record scores (%), median (IQR)	16.5 (17.8)	13.0 (12.0)	0.27 ^a^
Probing depth, *n* (%)			
<4 mm	8 (21.1%)	7 (19.4%)	0.04 ^b^
≥4 mm and <6 mm	23 (60.5%)	13 (36.1%)	
≥6 mm	7 (18.4%)	16 (44.4%)	
≥4-mm periodontal pocket with BOP, *n* (%)	15 (39.5%)	19 (52.8%)	0.35 ^c^
≥6-mm periodontal pocket with BOP, *n* (%)	3 (7.9%)	9 (25.0%)	0.06 ^c^
PISA (mm^2^), median (IQR)	47.2 (104.8)	52.7 (108.4)	0.82 ^a^
PESA (mm^2^), median (IQR)	1004.9 (367.5)	976.9 (455.6)	0.31 ^a^

IQR: Interquartile range. PISA: periodontal inflamed surface area. PESA: periodontal epithelial surface area. ^a^ Mann–Whitney U test. ^b^ χ^2^ test. ^c^ Fisher’s exact test. *p*-values < 0.05 were considered statistically significant.

**Table 5:** Logistic regression analysis with HHV-7 as dependent variable.

Clinical Variables	Odds Ratio	95% Confidence Interval	*p*-Value
Remaining teeth	0.95	0.87–1.03	0.19
Probing depth	1.32	0.60–2.93	0.49
≥6-mm periodontal pocket with BOP	2.92	0.57–15.0	0.20

## Data Availability

All data generated or analyzed in this study are included in this manuscript.

## Supplementary Materials

The following supporting information can be downloaded at https://www.mdpi.com/article/10.3390/life13020324/s1: Figure S1: Standard curve indicating HHV-6-positive DNA sample vs. CT values. Figure S2: Standard curve indicating HHV-7-positive DNA sample vs. CT values.
