# Supplementary figures and images for: Associations between Oral Human Herpesvirus-6 and -7 and Periodontal Conditions in Older Adults
Source: Life (Basel). 2023 Jan 23;13(2):324. doi: 10.3390/life13020324 (PMC9965650; doi:10.3390/life13020324)

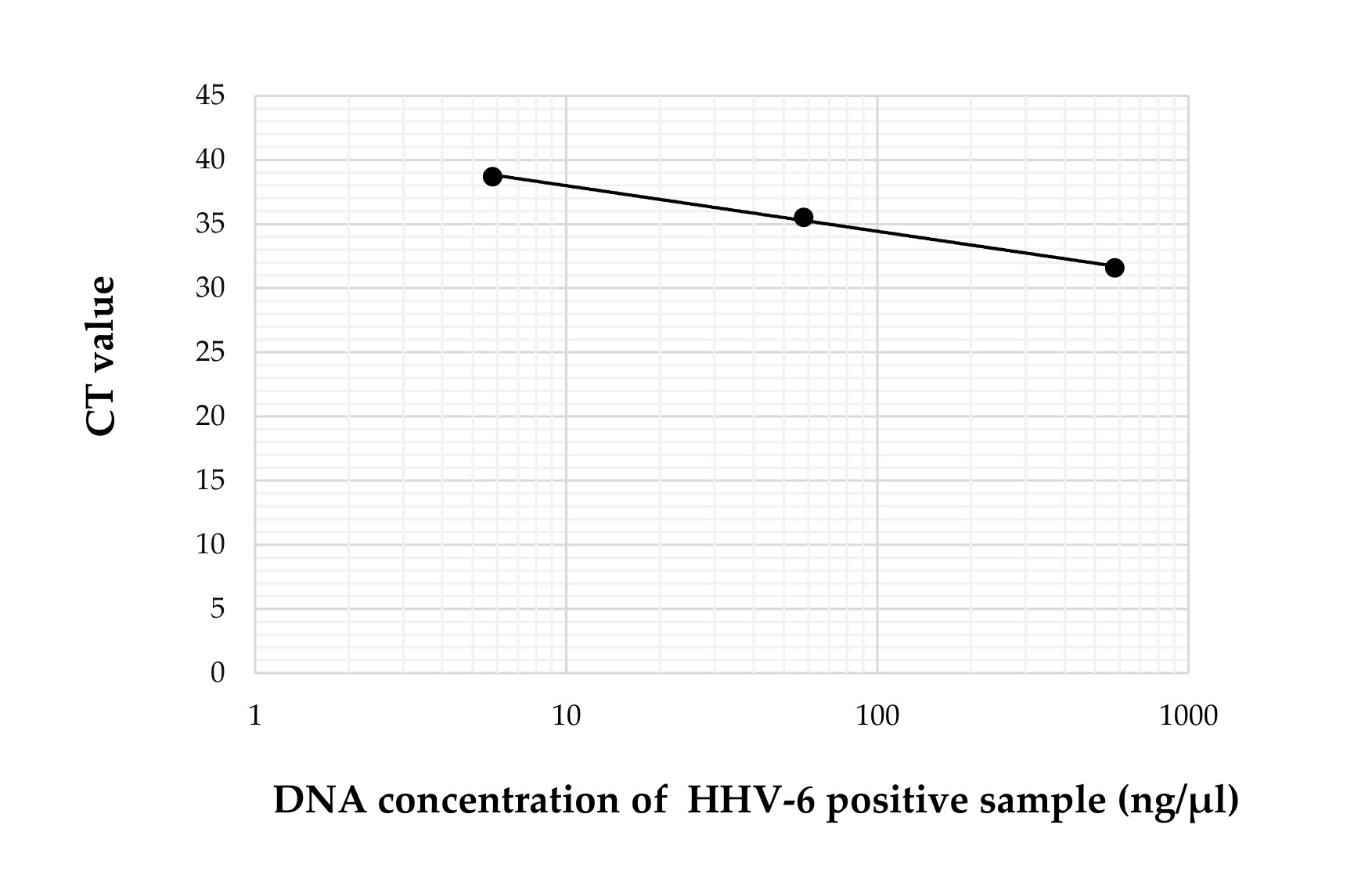


Figures S1


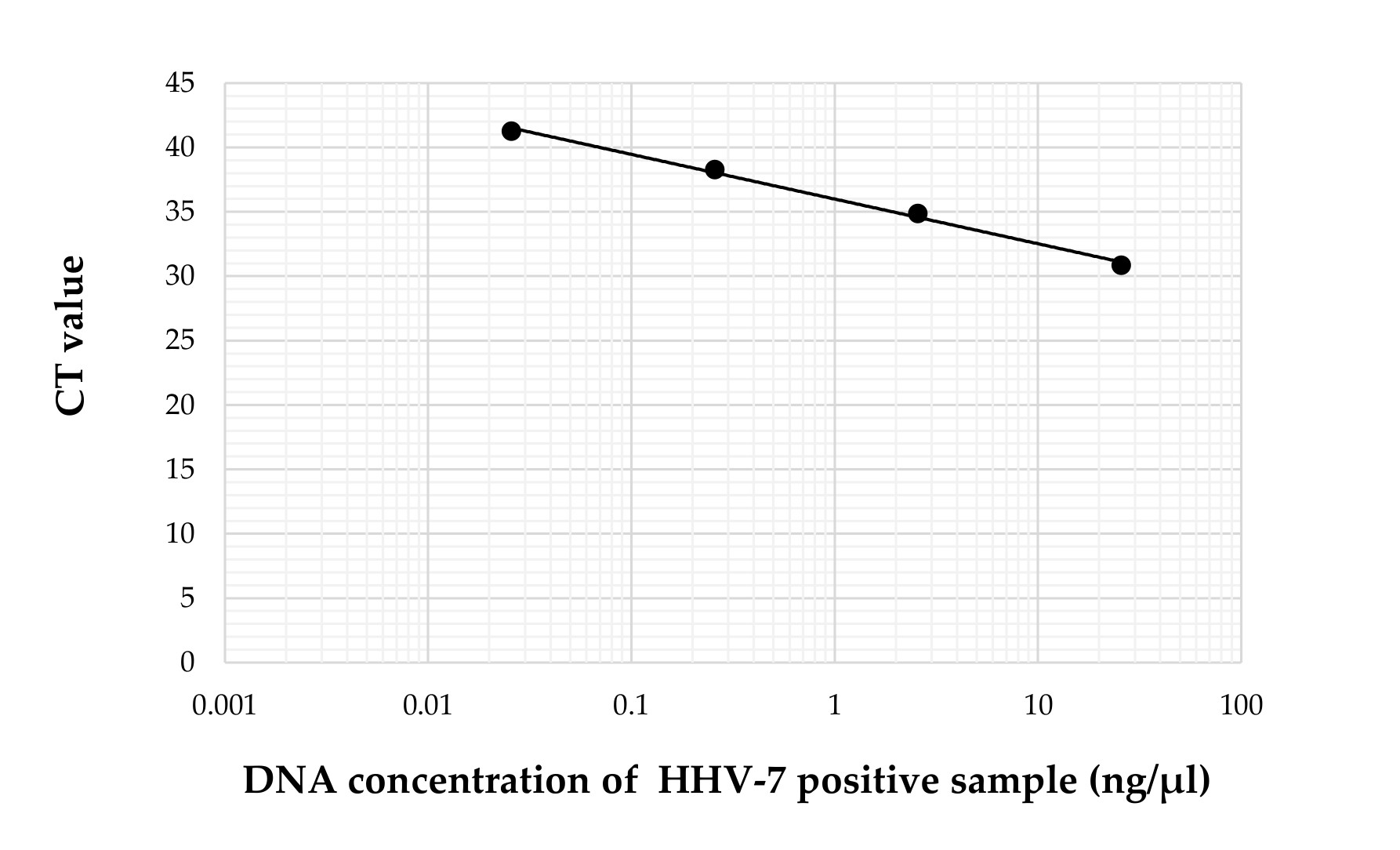


Figures S2

Supplement: Supplementary file 1 [file life-13-00324-s001.zip › life-2099969-supplementary.docx]
